# Structural and Electronic Properties of Iron(0) PNP Pincer Complexes

**DOI:** 10.1002/zaac.202100015

**Published:** 2021-05-04

**Authors:** Mathias Glatz, Nikolaus Gorgas, Berthold Stöger, Ernst Pittenauer, Liliana Ferreira, Luis F. Veiros, Maria José Calhorda, Karl Kirchner

**Affiliations:** ^1^ Institute of Applied Synthetic Chemistry Vienna University of Technology Getreidemarkt 9/163-AC 1060 Vienna Austria; ^2^ X-ray Center Vienna University of Technology Getreidemarkt 9/163-OC 1060 Vienna Austria; ^3^ Institute of Chemical Technologies and Analytics Vienna University of Technology Getreidemarkt 9 A-1060 Vienna Austria; ^4^ Department of Physics University of Coimbra 3004-516 Coimbra Portugal; ^5^ BioISI-Biosystems and Integrative Sciences Institute Faculdade de Ciências Universidade de Lisboa 1749-016 Lisboa Portugal; ^6^ Centro de Química Estrutural and Departamento de Engenharia Química Instituto Superior Técnico Universidade de Lisboa Av Rovisco Pais 1049-001 Lisboa Portugal

**Keywords:** Iron Complexes, PNP Pincer Ligands, Carbon Monoxide, DFT calculations

## Abstract

In the present work we have prepared and fully characterized several Fe(0) complexes of the type [Fe(PNP)(CO)_2_] treating Fe(II) complexes [Fe(PNP)(Cl)_2_] with KC_8_ in the presence of carbon monoxide. While complexes [Fe(PNP^NMe^‐*i*Pr)(CO)_2_], [Fe(PNP^NEt^‐*i*Pr)(CO)_2_] adopt a trigonal bipyramidal geometry, the bulkier and more electron rich [Fe(PNP^NH^‐*t*Bu)(CO)_2_] is closer to a square pyramidal geometry. Mössbauer spectra showed isomer shifts very close to 0 and similar to those reported for Fe(I) systems. Quadrupole splitting values range between 2.2 and 2.7 mm s^−1^ both in experiments and DFT calculations, while those of Fe(I) complexes are much smaller (∼0.6 mm s^−1^).

## Graphical abstract

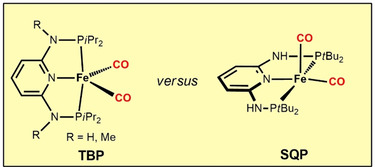

## Introduction

**Scheme 1:**
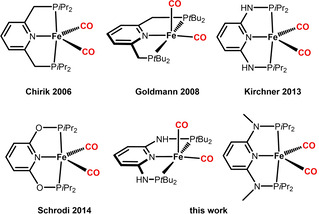
Pyridine‐based Iron(0) PNP Pincer Dicarbonyl Complexes.

Neutral pyridine‐based PNP pincer ligands are widely utilized in transition metal chemistry due to their combination of stability, activity and variability.^[1]^ They typically enforce a *meridional* κ^3^‐*P,N,P* coordination mode provided that three coordination sites are accessible at the metal center. We^[2]^ and others[^3^, ^4^, ^5^] reported recently the preparation and characterization of iron(0) PNP pincer complexes of the type [Fe(PNP)(CO)_2_] (Scheme 1). These complexes are typically orange or red solids with a low spin‐d^8^ configuration and, as expected, adopt a trigonal bipyramidal (TBP) geometry.^[6]^ The only exception was [Fe(PNP^CH2^‐*t*Bu)(CO)_2_] (PNP^CH2^‐*t*Bu=bis(di‐*tert*‐butylphosphinomethyl)pyridine) described by Goldman and co‐workers^[4]^ where the coordination geometry around the iron center was found to be closer to a square pyramidal (SQP) geometry.

Another unusual structural feature is that the CO ligand in the apical position of the SQP deviates significantly from linearity with an Fe−C−O angle of 171.9(1)°. Moreover, the spectroscopic properties of this complex are different from related Fe(0) PNP pincer complexes. First of all, the color of this compound is bright blue both in solution and in the solid state. Secondly, NMR spectra are typically very broad and not well resolved which is unexpected for a diamagnetic d^8^ low‐spin complex. As possible explanation it was suggested that [Fe(PNP^CH2^‐*t*Bu)(CO)_2_] undergoes a reversible interconversion between the SQP and TBP forms.

Here we are focusing on the synthesis and characterization of iron(0) complexes of the type [Fe(PNP)(CO)_2_] containing PNP pincer ligands based on the 2,6‐diaminopyridine scaffold where the aromatic pyridine ring and the phosphine moieties are connected via NH, N‐alkyl linkers.[^1g^, ^7^, ^8^, ^9^] We discuss structural and electronic aspects of these compounds and compare these with the known complexes described in Scheme 1.

## Results and Discussion

**Scheme 2:**
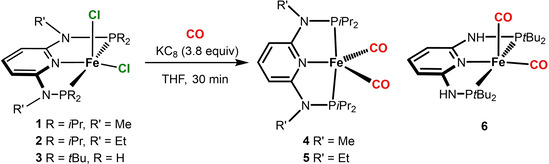
Synthesis of Fe(0) PNP complexes of the type [Fe(PNP)(CO)_2_].

The synthesis of Fe(0) complexes of the type [Fe(PNP)(CO)_2_] was achieved by stirring [Fe(PNP^NMe^‐*i*Pr)(Cl)_2_] (**1**), [Fe(PNP^NEt^‐*i*Pr)(Cl)_2_] (**2**), and [Fe(PNP^NH^‐*t*Bu)(Cl)_2_] (**3**) in THF with an excess of KC_8_ in the presence of carbon monoxide yielding [Fe(PNP^NMe^‐*i*Pr)(CO)_2_] (**4**), [Fe(PNP^NEt^‐*i*Pr)(CO)_2_] (**5**), and [Fe(PNP^NH^‐*t*Bu)(CO)_2_] (**6**), respectively, in 98, 94 and 91 % isolated yields (Scheme 2). All compounds are air‐sensitive but thermally stable orange to red solids. Complexes **4** and **5** were characterized by ^1^H, ^13^C{^1^H} and ^31^P{^1^H} NMR, IR spectroscopy, and elemental analysis. Surprisingly, complex **6** was NMR silent (*vide infra*) and its identity was established by IR spectroscopy, and elemental analysis. In addition, the molecular structures of complexes **4** and **6** were determined by X‐ray crystallography.

In the IR spectrum, two intense carbonyl bands are observed in the range of 1858 to 1801 cm^−1^. For comparison, in the related Fe(0) complexes [Fe(PNP^CH2^‐*i*Pr)(CO)_2_] (PNP^CH2^‐*i*Pr=bis(di‐*iso*‐propylphosphinomethyl)pyridine) these bands are found at 1842 and 1794 cm^−1^. The shift of the CO bands to somewhat higher frequencies is consistent with a less electron rich Fe(0) center in **4**–**6** as compared to [Fe(PNP^CH2^‐*i*Pr)(CO)_2_], which is apparently the stronger π base to the coordinated CO. In the ^13^C{^1^H} NMR spectrum the CO ligands give rise to a low‐field resonance triplet centered in the range at about 220 ppm with a coupling constant *J*_CP_ of 28 Hz. In the ^31^P{^1^H} NMR spectrum singlets at 181.7 and 183.7 ppm, respectively, were observed.

**Figure 1:**
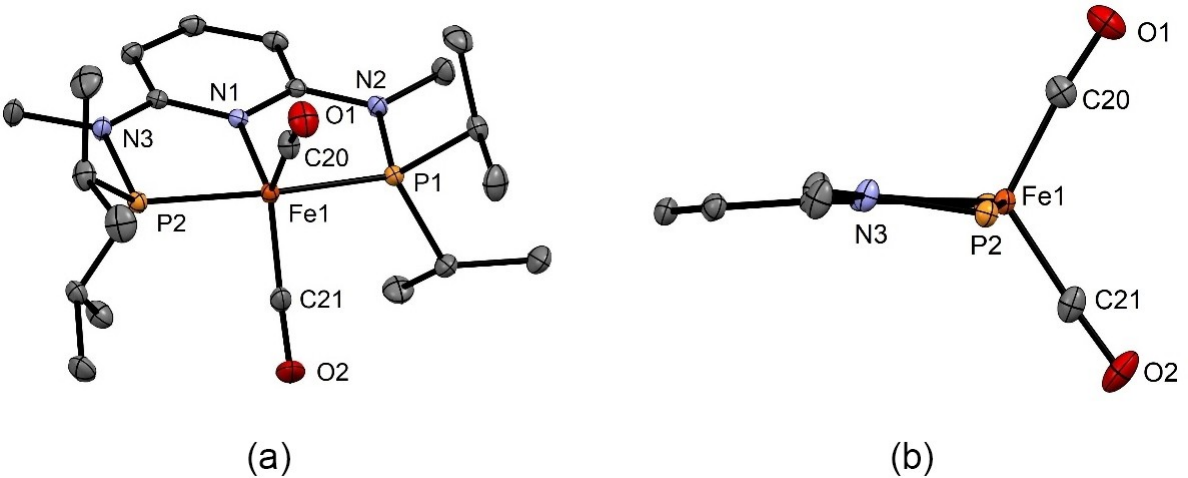
(a) Structural view of [Fe(PNP^NMe^‐*i*Pr)(CO)_2_] (**4**) showing 50 % thermal ellipsoids (H atoms omitted for clarity). (b) Inner part of **4** showing the trigonal bipyramidal structure. Selected bond lengths (Å) and bond angles (°): Fe1−P1 2.1717(4), Fe1−P2 2.1669(4), Fe1−N1 2.0189(9), Fe1−C20 1.749(1), Fe1−C21 1.740(1), P1−Fe1−P2 165.14(1), C20−Fe1−C21 119.54(6), Fe1−C20−O1 174.1(1), Fe1−C21−O2 176.9(1).

**Figure 2:**
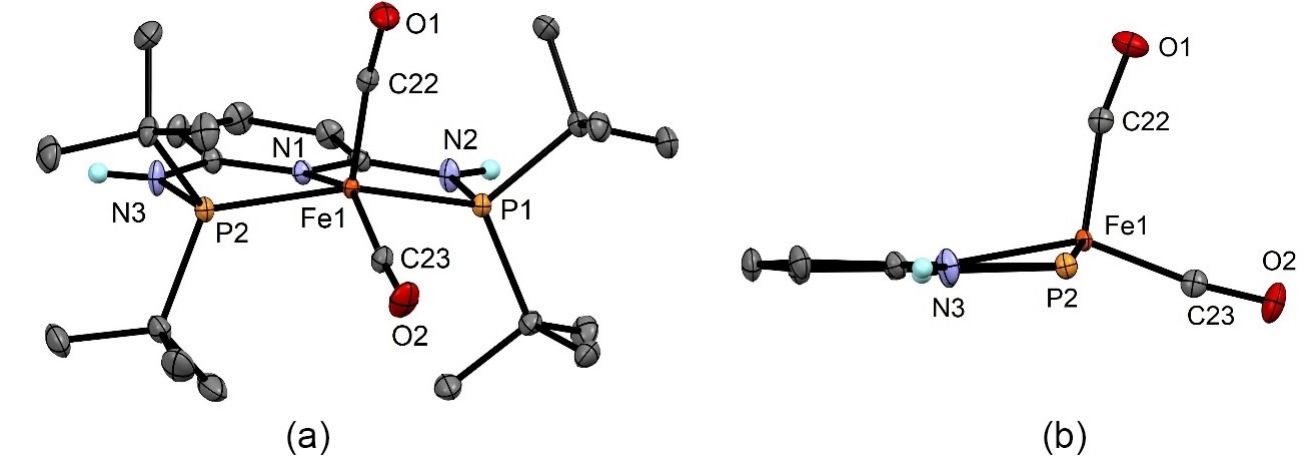
(a) Structural view of [Fe(PNP^NH^‐tBu)(CO)_2_]⋅3/4 (acetone) (6⋅3/4 (acetone)) showing 50 % thermal ellipsoids (most H atoms, solvent molecules, and a second independent complex omitted for clarity). (b) Inner part of 6 showing the square pyramidal structure as well as the significant bending of the apical CO ligand. Selected bond lengths (Å) and bond angles (°): Fe1−P1 2.2235(3), Fe1−P2 2.2206(3), Fe1−N1 2.0374(9), Fe1−C22 1.746(2), Fe1−C23 1.716(1), P1−Fe1−P2 156.29(2), C22−Fe1−C23 103.09(6), Fe1−C22−O1 167.18(8), Fe1−C23−O2 172.1(1).

Structural views of **4** and **6** are depicted in Figures 1 and 2 with selected bond distances and angles reported in the captions. In the case of **4**, the overall geometry about the iron center is best described as distorted trigonal bipyramidal, while the coordination geometry of the bulkier complex **6** is much closer to a SQP geometry as shown in Figure 2 than to the TBP geometry. For comparison, also the related complexes [Fe(PNP^NH^‐*i*Pr)(CO)_2_]^[2]^ and [Fe(PNP^CH2^‐*i*Pr)(CO)_2_]^[3]^ adopt a trigonal bipyramidal structure, whereas [Fe(PNP^CH2^‐*t*Bu)(CO)_2_]^[4]^ exhibits a square pyramidal geometry. In complex **4**, two Fe−C−O angles are almost linear with Fe1‐20‐O1 and Fe1−C21−O2 being 174.1(1) and 176.9(1) Å, respectively. In complex **6**, the Fe−C−O angles, in particular the one with the apical CO ligand, deviate significantly from linearity with Fe1−C22−O1 and Fe1−C23−O2 being 167.18(8) and 172.1(1) Å. The DFT calculated value of the apical CO ligand is 169.5°, clearly showing that this is not a packing but an electronic effect. A similar bending of the apical CO ligand was also observed in [Fe(PNP^CH2^‐*t*Bu)(CO)_2_].^[4]^

**Figure 3:**
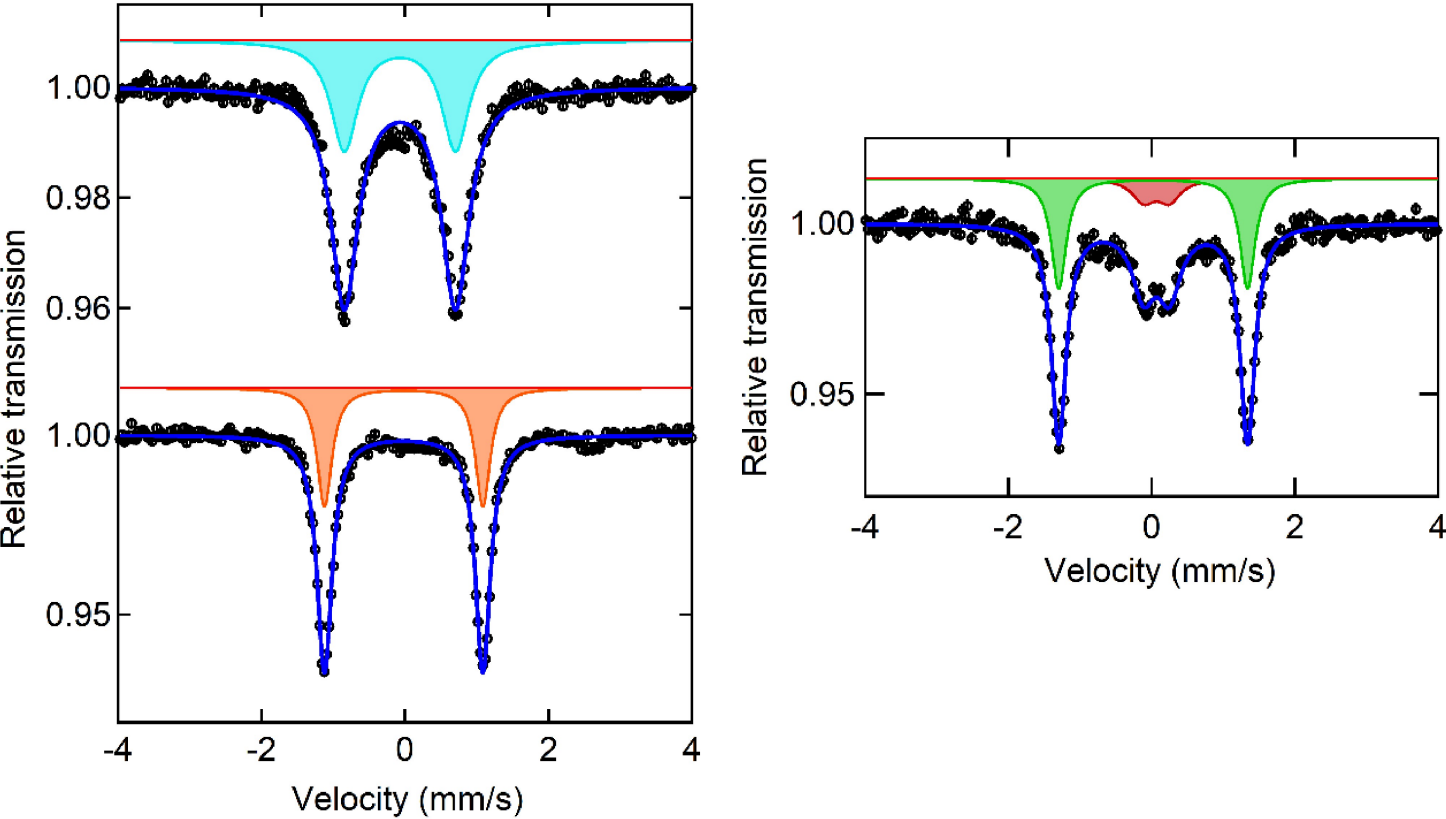
(right) ^57^Fe Mössbauer spectra of [Fe(PNP^NMe^‐*i*Pr)(CO)_2_] (**4**) (left, top) and [Fe(PNP^NEt^‐*i*Pr)(CO)_2_] (**5**) (left, bottom) and [Fe(PNP^NH^‐*t*Bu)(CO)_2_] (**6**) (right) collected at 78 K.

The ^57^Fe Mössbauer spectra of complexes **4**, **5** and **6** were obtained to further evaluate their electronic structure (Figure 3). The 78 K Mössbauer spectra of **4** and **5** are well‐fit to a major species (ca. 87 % and 94 % of iron) with parameters IS=−0.067 mm s^−1^ and QS=1.579 mm s^−1^ (**4**) and IS=−0.018 mm s^−1^ and QS=2.207 mm s^−1^ (**5**). On the other hand, two signals are clearly observed for complex **6**, with IS=0.021 mm s^−1^ and QS=2.635 mm s^−1^ (69 %) and IS=0.067 mm s^−1^ and QS=0.36 mm s^−1^ (31 %). The isomer shift values for all complexes are low, very similar and can be attributed to Fe(0) or Fe(I). There are not many reported Mössbauer parameters for iron complexes in low oxidation states. One example is the [Fe(^iPr^PDI)(CO)_2_]^+^ cation with IS=0.03 mm s^−1^, QS=0.62 mm s^−1^, which was attributed to Fe(I).^[10]^ The quadrupole splittings, on the contrary, display rather different values for the two species in **6** and an intermediate value in case of **4**.

One possible explanation for the presence of two species in **6** would be the possibility of a spin crossover, leading to a population of the triplet state in addition to the singlet ground state.

**Figure 4:**
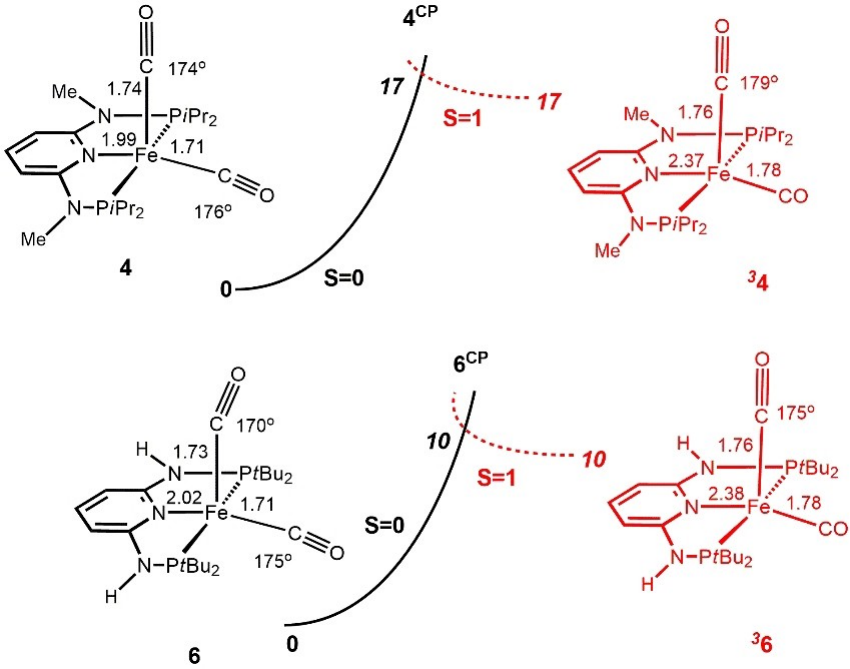
Fee energy profile (OPBE) for the spin state changes of complexes **4** and **6** (free energies in kcal mol^−1^). The black curves correspond to the spin‐singlet PES (*S*=0), and the red curve to the spin‐triplet PES (*S*=1).

A possible equilibrium between low spin (singlet, *S*=0) and high spin (triplet, *S* =1) isomers of complexes **4** and **6** was explored by means of DFT calculations^[11]^ and the resulting profiles are represented in Figure 4. For both complexes the singlet is the most stable spin state, while the triplet species are unstable, having the same free energies as the respective crossing point (**4^CP^** and **6^CP^**, see Computational details). According to these results, there should be no high spin species in equilibrium with the singlet isomers, neither for complex **4**, nor for complex **6**.

The possible existence of a TBP structure for **6** was also tested. However, all calculations led to observed SQP structure and attempts at generating a TBP‐type potential energy minimum were unsuccessful. Moreover, interconversion between TBP and SQP isomers, and exchange between the inequivalent carbonyl ligands of **6**, is predicted to be very fast on the NMR time scale, in agreement with the observation of only one carbonyl peak in the ^13^C{^1^H} NMR spectrum of **6**.

Finally, we considered again the Mössbauer parameters, compared the IS and QS for the three Fe(0) samples and the reported Fe(I) complex and noticed that the ISs are extremely similar. However, the QS values are significantly different. Therefore we calculated the two parameters for the three complexes **4**, **5** and **6**, and for one Fe(I) complex with the same type of pincer ligand, [Fe(PNP^NH^‐*i*Pr)(CO)_2_]^+^ (**7^+^**),^[12]^ whose oxygen sensitivity prevented its Mössbauer study, using the ADF program^[13]^ (see Computational details). The calculated QS parameter and the s‐electron density (ρ) at the Fe nucleus are given in Table 1 with the experimental ones for an easy comparison.

**Table 1:** Experimentally determined IS and QS (mm s^−1^) for complexes **4**–**7^+^** and DFT calculated s‐electron density (ρ, au) and QS (mm s^−1^) for complexes **4**–**7^+^**.

Complex	IS (exp)	ρ (calc)	QS (exp)	QS (calc)
**4**	−0.067	13594.49650	1.579	2.327
**5**	−0.018	13594.51581	2.207	2.295
**6**	0.021	13594.41263	2.635	2.711
**6^+^**	0.067	13594.31573	0.360	0.674
**7^+^**	–	13594.31701	–	0.664

The observed IS values are very similar for all complexes and all are very close to zero, as expected for Fe(0) and Fe(I) complexes. This is reflected in the almost negligible changes of the s‐electron density at the Fe nucleus (ρ). For this reason the IS values were not calculated, though they could be obtained by Neese's method.^[14]^ These **5** complexes are generally unstable toward oxidation and difficult to measure. For this reason, there are no experimentally values for the Fe(I) cation **7^+^**. Notice however, that the calculated s‐electron electronic densities ρ of **6^+^** and **7^+^** are at least 0.01 au lower than the other three (**4**–**6**), thus consistent with a higher oxidation state. The calculated QS are in a very good agreement for **5** and **6**, and not so good for **4**, but the values for **6^+^** and **7^+^** are significantly lower, decreasing from ∼2.3–2.7 to ∼0.67 mm s^−1^. The reported Fe(I) complex also has a QS=0.62 mm s^−1^. These results strongly suggest that the second signal observed in the Mössbauer spectrum of complex **6** results from its oxidation product, since **6** was modelled with loss of one electron (**6^+^**). It is likely that this is the first stage of oxidation, probably followed by decomposition.

## Conclusion

We have prepared Fe(0) complexes of the type [Fe(PNP)(CO)_2_] treating Fe(II) complexes [Fe(PNP)(Cl)_2_] with KC_8_ in the presence of carbon monoxide. While complexes [Fe(PNP^NMe^‐*i*Pr)(CO)_2_] (**4**), [Fe(PNP^NEt^‐*i*Pr)(CO)_2_] (**5**) adopt a trigonal bipyramidal geometry, the bulkier and more electron rich [Fe(PNP^NH^‐*t*Bu)(CO)_2_] (**6**) is closer to a square pyramidal geometry. Mössbauer spectra showed for complexes **4**–**6** isomer shifts very close to 0 and similar to that reported to Fe(I). However, quadrupole splitting values range between 2.2 and 2.7 mm s^−1^, both in experiments and DFT calculations, while those of Fe(I) complexes are much smaller (∼0.6 mm s^−1^). Therefore, the QS seems to be a better parameter for identification, though more work is needed. This was used to try and identify the impurity signal on the spectrum of **6** as its oxidation product **6^+^**. A possible equilibrium between low spin (singlet, *S*=0) and high spin (triplet, *S* =1) isomers of complexes **4** and **6** was explored by means of DFT calculations, but could be excluded.

## Experimental Section

**General Information**. All manipulations were performed under an inert atmosphere of argon by using Schlenk techniques or in a MBraun inert‐gas glovebox. The solvents were purified according to standard procedures.^[15]^ The deuterated solvents were purchased from Aldrich and dried over 4 Å molecular sieves. Complexes [Fe(PNP^NMe^‐*i*Pr)(Cl)_2_] (**1**),^[7]^ [Fe(PNP^NEt^‐*i*Pr)(Cl)_2_] (**2**),^[8]^ [Fe(PNP^NH^‐*t*Bu)(Cl)_2_] (**3**)^[9]^ and potassium graphite (KC_8_)^[16]^ were prepared according to the literature. ^1^H, ^13^C{^1^H}, and ^31^P{^1^H} NMR spectra were recorded on Bruker AVANCE‐250 and AVANCE‐400 spectrometers spectrometers. ^1^H and ^13^C{^1^H} NMR spectra were referenced internally to residual protio‐solvent, and solvent resonances, respectively, and are reported relative to tetramethylsilane (δ=0 ppm). ^31^P{^1^H} NMR spectra were referenced externally to H_3_PO_4_ (85 %) (δ=0 ppm).

The ^57^Fe Mössbauer spectra were recorded in transmission mode at 78 K using a conventional constant‐acceleration spectrometer and a 50 mCi ^57^Co source in a Rh matrix. The low temperature measurements were performed using a liquid nitrogen flow cryostat with a temperature stability of ±0.5 K. The velocity scale was calibrated using an α‐Fe foil. The spectra were fitted to Lorentzian lines using the WinNormos software program, and the isomer shifts reported are relative to metallic α‐Fe at room temperature.

All mass spectrometric measurements were performed on an Esquire 3000^*plus*^ 3D‐quadrupole ion trap mass spectrometer (Bruker Daltonics, Bremen, Germany) in positive‐ion mode by means of electrospray ionization (ESI). Mass calibration was done with a commercial mixture of perfluorinated trialkyl‐triazines (ES Tuning Mix, Agilent Technologies, Santa Clara, CA, USA). All analytes were dissolved in methanol “hypergrade for LC‐MS Lichrosolv” quality (Merck, Darmstadt, Germany) to form a concentration of roughly 1 mg/mL. Direct infusion experiments were carried out using a Cole Parmer model 74900 syringe pump (Cole Parmer Instruments, Vernon Hills, IL, USA) at a flow rate of 2 μL/min. Full scan and MS/MS (low energy CID)‐scans were measured in the range m/z 100–1100 with the target mass set to m/z 1000. Further experimental conditions include: drying gas temperature: 150 °C; capillary voltage: −4 kV; skimmer voltage: 40 V; octapole and lens voltages: according to the target mass set. Helium was used as buffer gas for full scans and as collision gas for MS/MS‐scans in the low energy CID mode. The activation and fragmentation width for tandem mass spectrometric (MS/MS, CID) experiments was set to 6 Da to cover the main isotope cluster for fragmentation. The corresponding fragmentation amplitude ranged from 0.4 to 0.6 V in order to keep a precursor ion intensity of low abundance in the resulting MS/MS spectrum. All mass calculations are based on the lowest mass (i. e. most abundant) iron isotope (^56^Fe‐isotope). Mass spectra and CID spectra were averaged during data acquisition time of 1 to 2 min and one analytical scan consisted of five successive micro scans resulting in 50 and 100 analytical scans, respectively, for the final full scan mass spectrum or MS/MS spectrum.

**Syntheses. [Fe(PNP^NMe^‐iPr)(CO)_2_] (4)**. [Fe(PNP^NMe^‐*i*Pr)(CO)(Cl)_2_] (**1**) (200 mg, 0.380 mmol) was added to a suspension of freshly prepared KC_8_ (200 mg, 1.479 mmol) in THF (10 mL) and CO was bubbled through the reaction mixture for 30 min, whereupon the solution changed from yellow to orange‐red. The solution was decanted from graphite and filtered through basic alumina. The filtrate was collected and the solvent was removed under vacuum affording **4** as an air‐sensitive orange solid. Yield: 190 mg (98 %). Anal Calc. for C_21_H_37_FeN_3_O_2_P_2_ (MW: 481.34) C, 52.40; H, 7.75; N, 8.73. Found: C, 52.29; H, 7.80; N, 8.80 %. ^1^H NMR (δ, C_6_D_6_, 20 °C):6.94 (tt, *J*=8.1, *J*=1.4 Hz, 1H, py^4^), 5.55 (d, *J*=8.1 Hz, 2H, py^3,5^), 2.40 (s, 6H, NMe), 2.37–2.25 (m, 4H, *i*Pr), 1.36 (dd, *J*=16.4, 7.4 Hz, 12H, *i*Pr), 1.11 (dd, *J*=13.9, 7.0 Hz, 12H, *i*Pr). ^13^C{^1^H} NMR (δ, C_6_D_6_, 20 °C): 219.6 (t, ^*3*^ *J_CP_* =28.0 Hz, CO), 161.2 (t, *J*=10.6 Hz, py^2,6^), 132.8 (s, py^4^), 94.9 (t, *J*=3.4 Hz, py^3,5^), 32.3 (d, *J*=3.0 Hz, NMe), 29.6 (t, *J*=11.0 Hz, *i*Pr), 17.5 (s, *i*Pr), 16.9 (t, *J*=3.3 Hz, *i*Pr).^31^P{^1^H} NMR (δ, C_6_D_6_, 20 °C): 181.7. IR (ATR, cm^−1^): 1856 (ν_CO_), 1802 (ν_CO_). ESI‐MS (m/z, THF); pos. ion: 481.3 [M]^+^, 453.4 [M−CO]^+^.

**[Fe(PNP^NEt^‐iPr)_2_(CO)_2_]) (5)**. This complex was prepared analogously to **4** using [Fe(PNP^NEt^‐*i*Pr)(Cl)_2_] (**2**) (200 mg, 0.381 mmol) and KC_8_ (200 mg, 1.479 mmol) as starting materials. Yield: 183 mg (94 %), orange solid. Anal Calc. for C_23_H_41_FeN_3_O_2_P_2_ (MW: 509.39): C, 54.23; H, 8.11; N, 8.25. Found: C, 54.39; H, 8.40; N, 8.21 %. ^1^H NMR (δ, C_6_D_6_, 20 °C): 6.89 (t, *J*=8.1 Hz, 1H, py^4^), 5.59 (d, *J*=8.1 Hz, 2H, py^3,5^), 2.98 (qd, *J*=6.9, 4.6 Hz, 4H, NEt), 2.26 (td, *J*=7.1, 3.8 Hz, 4H, *i*Pr), 1.42 (dd, *J*=16.5, 7.1 Hz, 12H, *i*Pr), 1.17 (dd, *J*=14.2, 7.0 Hz, 12H, *i*Pr), 0.86 (t, *J*=7.0 Hz, 6H, NEt). ^13^C{^1^H} NMR (δ, C_6_D_6_, 20 °C): 219.90 (t, *J*=27.5 Hz, CO), 160.63 (t, *J*=10.8 Hz, py^2,6^), 132.17 (s, py^4^), 95.82 (t, *J*=3.5 Hz, py^3,5^), 40.10 (s, NEt), 30.05 (t, *J*=11.0 Hz, *i*Pr), 17.84 (s, *i*Pr), 17.28 (t, *J*=3.5 Hz, *i*Pr), 12.90 (s, NEt). ^31^P{^1^H} NMR (δ, C_6_D_6_, 20 °C): 183.7. IR (ATR, cm^−1^): 1858 (ν_CO_), 1801 (ν_CO_). ESI‐MS (m/z, THF); pos. ion: 509.3 [M]^+^, 481.3 [M−CO]^+^.

**[Fe(PNP^NH^‐tBu)(CO)_2_]) (6)**. This complex was prepared analogously to **4** using [Fe(PNP^NH^‐*t*Bu)(Cl)_2_] (**3**) (200 mg, 0.381 mmol) and KC_8_ (170 mg, 1.259 mmol) as starting materials. Yield: 181 mg (91 %), red solid. Anal Calc. for C_23_H_41_FeN_3_O_2_P_2_ (MW: 509.39) C, 54.23; H, 8.11; N, 8.25. Found: C, 54.40; H, 8.00; N, 8.34. IR (THF, cm^−1^): 1865 (ν_CO_), 1814 (ν_CO_).

**X‐ray Structure Determination**. X‐ray diffraction data of **4** and **6** (CCDC 1949182, 1949182) were collected at *T*=100 K in a dry stream of nitrogen on a Bruker Kappa APEX II diffractometer system using graphite‐monochromatized Mo‐*K*α radiation (λ=0.71073 Å) and fine sliced *ϕ*‐ and *ω**‐*** scans. Data were reduced to intensity values with SAINT and an absorption correction was applied with the multi‐scan approach implemented in SADABS.17 The structures were solved by the dualspace method implemented in SHELXT^[18]^ and refined against *F* with Jana2006.^[19]^ Non‐hydrogen atoms were refined with anisotropic displacement parameters. The H atoms connected to C atoms were placed in calculated positions and thereafter refined as riding on the parent atoms. The H atoms connected to O and N were refined freely. Molecular graphics were generated with the program MERCURY.^[20]^

**Computational Details**. The computational results presented have been achieved in part using the Vienna Scientific Cluster (VSC). Calculations were performed using the Gaussian 09 software package^[21]^ and the OPBE functional without symmetry constraints. This functional combines Handy's OPTX modification of Becke's exchange functional^[22]^ with the gradient corrected correlation functional of Perdew, Burke, and Ernzerhof,^[23]^ and it was shown to be accurate in the calculation of spin state energy splitting for first transition row species.^[24]^ The optimized geometries were obtained with the Stuttgart/Dresden ECP (SDD) basis set^[25]^ to describe the electrons of Fe and a standard 6‐31G** basis set^[26]^ for the other atoms. The electronic energies were converted to free energy at 298.15 K and 1 atm by using zero‐point energy and thermal energy corrections based on structural and vibration frequency data calculated at the same level.

The Minimum Energy Crossing Points (**4^CP^** and **6^CP^**) are the points where the change of spin state occurs, and the system goes from the singlet (*S*=0) Potential Energy Surface (PES) to the triplet one (*S*=1), resulting in a spin‐forbidden process. In those points, both the energy as well as the geometry of both spin isomers are equal.[^27^, ^28^] They were determined using a code developed by Harvey *et al*.^[29]^ This code consists of a set of shell scripts and Fortran programs that uses the Gaussian results of energies and gradients of both spin states to produce an effective gradient pointing towards the crossing point. This is not a stationary point and, hence, a standard frequency analysis is not applicable. Therefore, the free energy values of the crossing points were obtained through frequency calculations projected for vibrations perpendicular to the reaction path.^[30]^ The value presented is the mean of the values obtained for both PES.

The DFT approach in the ADF program^[13]^ was used to calculate the Mössbauer parameters. The geometries were first optimized without symmetry constraints, considering solvent (tetrahydrofuran), with gradient correction, using the Vosko‐Wilk‐Nusair^[31]^ Local Density Approximation of the correlation energy and the Generalized Gradient Approximation with Becke's exchange^[32]^ and Perdew's^[33]^ correlation functionals. Unrestricted calculations were carried out for open shell complexes. The solvent correction was taken into account using the COSMO approach implemented in ADF. Relativistic effects were treated with the ZORA approximation.^[34]^ Frequency calculations showed that all structures corresponded to true minima and the ν_CO_ stretching frequencies were reproduced with a scale factor of 0.98. Quadruple ζ Slater‐type orbitals (STO) with a set of four polarization functions were used to describe all the electrons of all the elements. The attempts at obtaining IS values from a plot led to a scatter of points because all the values are too close to 0, and the exact density was therefore more informative.
